# Electroacupuncture ameliorates inflammatory response induced by retinal ischemia-reperfusion injury and protects the retina through the DOR-BDNF/Trkb pathway

**DOI:** 10.3389/fnana.2022.1057929

**Published:** 2023-01-05

**Authors:** Runjie Guo, Yongjie Zhang, Yue Geng, Ping Chen, Tiantian Fu, Yong Xia, Ren Zhang, Yuan Zhu, Jingling Jin, Nange Jin, Hong Xu, Xuesong Tian

**Affiliations:** ^1^Experiment Center for Science and Technology, Shanghai University of Traditional Chinese Medicine, Shanghai, China; ^2^School of Acupuncture-Moxibustion and Tuina, Shanghai University of Traditional Chinese Medicine, Shanghai, China; ^3^Shanghai Chinese Medicine Literature Museum, Shanghai, China; ^4^Shanghai Jinshan District Hospital of Traditional Chinese and Western Medicine, Shanghai, China; ^5^Division of Cancer Medicine, MD Anderson Cancer Center, Houston, TX, United States; ^6^Department of Vision Sciences, University of Houston College of Optometry, Houston, TX, United States; ^7^Department of Acupuncture-Moxibustion, Longhua Hospital, Shanghai University of Traditional Chinese Medicine, Shanghai, China

**Keywords:** retinal ischemia-reperfusion injury (RIRI), electroacupuncture (EA), δ-opioid receptor (DOR), inflammation, apoptosis

## Abstract

**Objectives:** Retinal ischemia-reperfusion injury (RIRI) is the common pathological basis of many ophthalmic diseases in the later stages, and inflammation is the primary damage mechanism of RIRI. Our study aimed to assess whether electroacupuncture (EA) has a protective effect against RIRI and to elucidate its related mechanisms.

**Methods:** A high-intraocular pressure (HIOP) model was used to simulate RIRI in Wistar rats. EA was applied to the EA1 group [Jingming (BL1) + Shuigou (GV26)] and the EA2 group [Jingming (BL1) + Hegu (LI4)] respectively for 30 min starting immediately after the onset of reperfusion and repeated (30 min/time) at 12 h and then every 24 h until days 7 after reperfusion. The pathological changes in the retina were observed by H and E staining after HIOP. Terminal deoxynucleotidyl transferase dUTP nick end labeling (TUNEL) staining was utilized to observe retinal cell apoptosis. The mRNA expression of IL1-β, TNF-α, IL-4, IL-10, δ-opioid receptor (DOR), brain-derived neurotrophic factor (BDNF), and tropomyosin-related kinase B (TrkB) in the retina was measured by quantitative real-time PCR.

**Results:** HIOP caused structural disorders of the retina, decreased RGCs, and increased retinal cell apoptosis. At 1 and 3 days of RIRI, retinal apoptotic cells in the EA group were significantly reduced, while there was no distinct difference in the EA group compared with the HIOP group at 7 days of RIRI. Compared with that in the HIOP group, the expression of anti-inflammatory factors, DOR and TrkB was increased, and the expression of pro-inflammatory factors was decreased in the EA group. In contrast, HIOP had no appreciable effect on BDNF expression.

**Conclusion:** EA at Jingming (BL1) and Shuigou (GV26) or at Jingming (BL1) and Hegu (LI4) may inhibit RIRI induced inflammation through activating the DOR-BDNF/TrkB pathway to protect the retina, especially the pair of Jingming (BL1) and Shuigou (GV26) has better inhibitory effects on inflammation.

## Introduction

Retinal ischemia-reperfusion injury (RIRI) is a condition in which the original function of the retina is not restored after retinal reperfusion subsequent to an ischemic state, structural damage is severe, and irreversible damage may even occur (Pan et al., 2014). RIRI is the common pathological basis of many ophthalmological diseases, such as diabetic retinopathy (DR), retinal vein occlusion (RVO), and glaucoma. It is also the leading cause of vision loss or visual impairment (Hartsock et al., 2016). Many factors work together in the pathological process of RIRI, such as inflammation, oxidative stress, glutamate excitotoxicity, and blood-eye barrier destruction (Osborne et al., 2004). Among them, inflammation plays an essential role in this process. Therefore, researching how to inhibit the inflammatory response caused by RIRI and finding the key target of RIRI is of great significance for its treatment.

δ-Opioid receptor (DOR), as an oxygen-sensitive protein sensitive to ischemia and hypoxia, is widely distributed in the retina and is primarily expressed in the nerve fiber layer (NFL), ganglion cell layer (GCL) and inner plexiform layer (IPL; Husain et al., 2009). Another study showed that activating DOR reduced the expression of TNF-α after RIRI in rats, and inhibition of DOR reversed this effect (Husain et al., 2011). Peng et al. (2009) found that activation of DOR corrected the redox balance after RIRI and played a crucially protective role in RIRI. In previous research by our group, the use of the DOR agonist Tan67 to activate DOR in brain I/R effectively increased the expression level of the anti-inflammatory factor IL-10 and reduced the expression level of the pro-inflammatory factor IL-1β (Geng et al., 2020). Furthermore, recent evidence has also shown that DOR can protect ischemic and hypoxic neurons in various ways, such as by maintaining ion balance, reducing oxidative stress damage, inhibiting excitotoxicity, and inhibiting inflammation (Estrada et al., 2016; Min et al., 2018).

Brain-derived neurotrophic factor (BDNF) is a type of neurotrophic factor. A study has shown that BDNF can bind to tropomyosin-related kinase B (TrkB) with high affinity to protect RGCs and save retinal function (Osborne et al., 2018). An increasing number of studies have found that electroacupuncture (EA), an extended technique based on traditional Chinese acupuncture combined with current electrotherapy, is closely related to anti-inflammatory effects (Lan et al., 2013; Liu et al., 2015; Shen et al., 2016; Jiang et al., 2017). Pagani et al. (2006) found that EA might exert its action on the regulation of retinal nerve growth factor (NGF) and BDNF and/or their receptors in retinal cells. Our research team has also proven that EA can activate DOR to exert anti-inflammatory effects through the BDNF/TrkB pathway and alleviate cerebral ischemia-reperfusion injury (Geng et al., 2020). However, whether DOR activation in rats with RIRI can inhibit the induced inflammatory response *via* BDNF/TrkB to exert neuroprotection is unclear.

Hence, in this investigation, we utilized high intraocular pressure (HIOP) to create a RIRI model in rats and selected two pairs of acupuncture points: (1) Jingming (BL1) and Shuigou (GV26); and (2) Jingming (BL1) and Hegu (LI4), for EA. We explored whether these points have protective effects and investigated the underlying mechanisms.

## Materials and Methods

### Animals

Adult male Wistar rats (250–280 g, 6–8 weeks old) were purchased from the Shanghai Laboratory Animal Company (Shanghai, China) and bred in the Experimental Animal Center of Shanghai University of Traditional Chinese Medicine. The rats were housed in cages and allowed free access to feed and water. The holding room possessed a 12-h light/dark cycle, 60%–70% relative humidity, and a temperature of 22°C ± 2°C. The animals were allowed a fortnight to adapt before experimentation. All experiments in this study were compiled to the guidelines for the welfare of animals, and the animal protocols were permitted by the Experimental Animal Ethics Committee of Shanghai University of Traditional Chinese Medicine (PZSHUTCM200731001).

### Establishment of the HIOP model

The animal model was prepared following a reported method with modifications (Hartsock et al., 2016). In brief, rats were anesthetized *via* inhalation of 2.5% isoflurane and maintained under anesthesia with 1.5% isoflurane/oxygen. The pupils were dilated with 1% tropicamide (Bausch & Lomb, New York, USA), and the corneas were anesthetized topically with 0.5% tetracaine hydrochloride eye drops (Bausch & Lomb, New York, USA). The anterior chamber of the left eye was inserted with a 33-gauge needle (JBP Co. Tokyo, Japan). The pressure of the left eye was increased and maintained at 110 mmHg by adding normal saline. The intraocular pressure was maintained for 60 min and then slowly decreased to normal. Rats were sacrificed at 24, 72, 120, or 168 h after the procedure.

### Experimental groups and EA treatment

**Figure 1:**
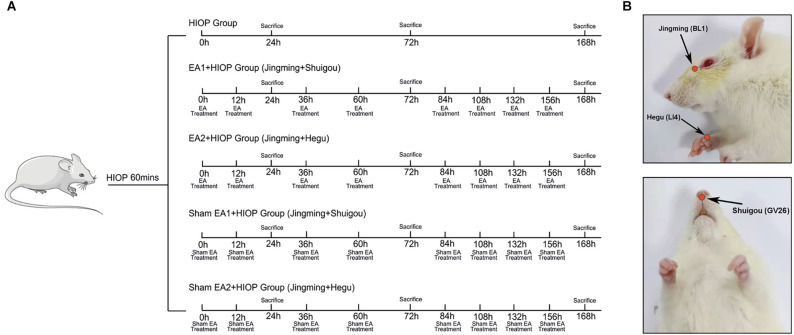
**(A)** Detailed acupuncture time and frequency. **(B)** Acupoints diagram of a rat.

Healthy rats were randomly assigned into the following seven groups: (1) the control group (Control); (2) the sham group (Sham); (3) the HIOP group (HIOP); (4) the EA1 [Jingming (BL1) + Shuigou (GV26)] + HIOP group (EA1); (5) the EA2 [Jingming (BL1) + Hegu (LI4)] + HIOP group (EA2); (6) the sham EA1 [Jingming (BL1) + Shuigou (GV26)] + HIOP group (SEA1); and (7) the sham EA2 [Jingming (BL1) + Hegu (LI4)] + HIOP group (SEA2). The control group was not subjected to any treatment. In the sham group, the 33-gauge needle was inserted into the anterior chamber for 1 min and removed without elevating the pressure. In the HIOP group, the RIRI model was established without any treatment. The EA1 group underwent EA stimulation on the ipsilateral side at Jingming (BL1) and Shuigou (GV26). The EA2 group underwent EA stimulation on the ipsilateral side at Jingming (BL1) and the contralateral side at Hegu (LI4). EA was applied immediately after the onset of reperfusion and repeated (30 min/time) at 12 h and then every 24 h until day 7 after reperfusion. Because Jingming (BL1) is around the eyes, we select Jingming acupoint on the ipsilateral side according to the principle of “neighboring acupoint selection” in traditional Chinese medicine. Hegu (LI4) acupoint is far away from the eyes. According to the principle of “treat disease in one side of the body by needling points on the opposite side” in traditional Chinese medicine, the contralateral Hegu (LI4) is selected for EA treatment. For selecting acupoints EA1 and EA2, we refer to the animal acupuncture points map published by the Experimental Acupuncture–Moxibustion Research Association of China. The EA apparatus (SDZ-V EA, Suzhou, China) was set as a mode of disperse-dense waves with a frequency of 4/20 Hz and currents of 1–2 mA. 4/20 Hz refers to the combination of disperse-dense waves alternating with 4 Hz sparse waves and 20 Hz dense waves. 1–2 mA refers to intensity initially set at 1 mA, then increased stepwise to 2 mA, and each lasted for 15 min. In the experiment, the intensity of 1–2 mA made the rat’s beard or muscle shake slightly without hissing and struggling. Based on the premise of experimental animal tolerance, stepwise increasing of EA intensity is a commonly used method in basic research (Qi et al., 2016). In the SEA1 and SEA2 groups, the needles were punctured superficially beside each acupoint, which corresponded to EA1 and EA2 without electrical stimulation. Figure 1 shows the detailed EA timing and frequency.

### Hematoxylin and eosin staining

The rats were sacrificed on schedule. The eyeballs were taken out and fixed with Davidson’s fixative overnight. Next, the eyes were fixed with 4% paraformaldehyde for 3 days prior to paraffin embedding. Then, each eye globe was randomly divided into 4-mm-thick sections through the optic nerve of each eye, and the sections were stained with hematoxylin and eosin (HE). The observations and measurements were performed under a light microscope (Olympus, Japan). The total thickness of the retina (from the ganglion cell layer (GCL) to the photoreceptor layer (PL) and each layer of the retina) were estimated in four adjoining areas with a 1 mm distance to the optic nerve center.

### TUNEL staining

The terminal deoxynucleotidyl transferase dUTP nick end labeling (TUNEL) kit (Beyotime Biotechnology, Shanghai, China) is used to detect apoptotic cells in the retina. Eye sections were deparaffinized, rehydrated, and then incubated in a 20 μg/ml proteinase K (Beyotime Biotechnology, Shanghai, China) working solution for 15 min at room temperature. After rinsing with phosphate-buffered saline three times for 5 min each, the sections were stained following the specification. Finally, the retinal cell nucleus was labeled with Hoechst 33342 (Beyotime Biotechnology, Shanghai, China). The immunofluorescence images were detected by fluorescent confocal microscopy (Leica, Germany) at 400× magnification. The TUNEL-positive cells were green, and the retinal cell nuclei were blue. Five random fields per section were imaged to observe the number of apoptotic cells.

### RNA extraction and qRT-PCR

The rats were sacrificed on schedule, and the retinal tissue was peeled from the eyes and rapidly frozen in liquid nitrogen. Total RNA was isolated from the tissue by using TRIzol reagent (Takara, Shiga, Japan) and incubated with chloroform for 15 min. After 15 min of centrifugation at 12,000× *g* 4°C, isopropanol was added to it and the admixture underwent a centrifugation at 12,000× *g* at 4°C for 15 min. Then, 75% ethanol prepared with RNase-free water was used to wash the RNA, and diethyl pyrocarbonate water resuspended the RNA. Afterward, cDNA was synthesized with PrimeScript RT reagent kit (Takara, Shiga, Japan) at 37°C for 15 min followed by 85°C for 5 s. qRT-PCR was performed by SYBR PrimeScript Plus RT-PCR Kit (Takara, Shiga, Japan) according to the operating instructions and applied with ABI 7500 PCR system (ABI, USA). The reaction was performed with 40 amplification cycles of denaturation at 95°C for 30 s followed by 94°C for 5 s and 60°C for 34 s. The quantity of target mRNA in test samples was detected and normalized to β-actin. The 2^−ΔΔct^ method was used to quantify the relative gene expression. Each sample was tested in triplicate. The Table 1 displays the sequences of the primers applied in this study.

**Table 1:** Primer information in this research.

Gene	Forward (5’-3’)	Reverse (5’-3’)
IL-4	TGTCACCCTGTTCTGCTTTC	CCTGGTTCAAAGTGTTGATGA
IL-10	AGGGTTACTTGGGTTGCCAA	TCAGCTTCTCTCCCAGGGAA
IL-1β	TCAAGCAGAGCACAGACCTG	GAAGACACGGGTTCCATGGT
TNF-α	CCGTGGGTTGGACAGATGAA	TGATTGCCCCGCTTACAGTT
DOR	GGACGCTGGTGGACATCAAT	CGTAGAGAACCGGGTTGAGG
BDNF	CAATGCCGAACTACCCAATC	CTTATGAACCGCCAGCCAAT
TrkB	CTTCAGTGGTTCTACAACGGA	ATATGAGTGGGGTTATCCAGC
β-actin	CCTCTATGCCAACACAGT	AGCCACCAATCCACACAG

### Data and statistical analysis

All data in this research are presented as the mean ± standard error of the mean (SEM). SPSS software (version 23; IBM Corp., Armonk, NY, USA) was applied to conduct statistical analysis. Comparisons between the two groups were assessed using Student’s *t*-tests. Multiple-group comparisons were performed using one-way ANOVA followed by *post hoc* comparisons with Dunnett’s *post-hoc* test and Fisher’s LSD test. When the equal variance assumption was not met, the data were compared using nonparametric tests. For analysis of each experiment, a value of *P* < 0.05, *P* < 0.01, and *P* < 0.001 was deemed a significant difference.

## Results

### RIRI can cause retinal degeneration and retinal cell apoptosis

**Figure 2:**
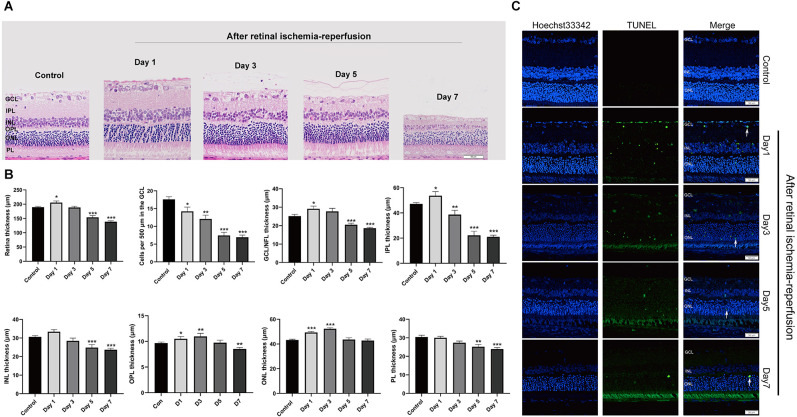
HIOP was used to cause RIRI in rats. **(A)** After retinal ischemia-reperfusion for 1, 3, 5, and 7 days, H&E staining of retinal cross-sections showed degeneration of RGCs and a decrease in the thickness of retinal tissue. GCL, ganglion cell layer; INL, inner nuclear layer; IPL, inner plexiform layer; ONL, outer nuclear layer; OPL, outer plexiform layer; PL, photoreceptor layer (*n* = 5). Scale bar: 50 μm. **(B)** Measurement of retinal thickness from the ganglion cell layer (GCL) to the photoreceptor layer (PL); the number of cells per 500 μm in the GCL and the mean thicknesses of different retinal layers in retinal ischemia-reperfusion rats at 1, 3, 5, and 7 days are shown. **(C)** Apoptotic cells were detected by TUNEL assay in retinal sections 1, 3, 5, and 7 days after ischemia-reperfusion injury in rats. Representative images of TUNEL-positive cells (green) are shown. The nuclei were counterstained with Hoechst 33342 (blue). Scale bar: 50 μm. The data are presented as the mean ± SEM (*P < 0.05, **P < 0.01, ***P < 0.001. *n* = 5 for each group). HIOP, high-intraocular pressure; RIRI, retinal ischemia-reperfusion injury; TUNEL, terminal deoxynucleotidyl transferase dUTP nick end labeling.

First, we induced acute RIRI in rats by HIOP. H&E staining was used to show the histopathological changes in the retina after retinal ischemia-reperfusion for 1, 3, 5, and 7 days (Figure 2A). We obtained the thickness of each layer of the retina and the number of retinal ganglion cells (RGCs) by measuring the HE images. According to statistics, on the first day of retinal ischemia-reperfusion, the overall thickness of each layer of the retina showed an increasing tendency. However, at 3, 5, and 7 days of retinal ischemia-reperfusion, the overall thickness of each layer of the retina began to exhibit a degressive trend. RGCs gradually decreased from the first to the 7th day (Figure 2B). Apoptotic cells in the retina were detected with a TUNEL assay in retinal sections after retinal ischemia-reperfusion for 1, 3, 5, and 7 days. We found that the numbers of apoptotic cells on the first and third days were significantly greater than those on the fifth and seventh days, and apoptotic cells gradually emerged from the GCL to the inner nuclear layer (INL; Figure 2C).

### EA can ameliorate injury and apoptosis of the retina at 1 day of RIRI in rats

**Figure 3:**
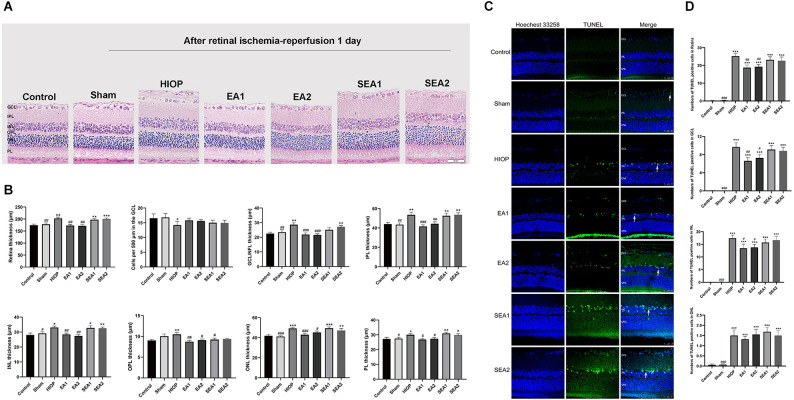
EA can ameliorate injury and apoptosis of the retina at 1 day of RIRI in rats. **(A)** H&E staining of retinal cross-sections showed the retinal tissue thickness of ischemia-reperfusion (I/R) rats in every group at 1 day of reperfusion. GCL, ganglion cell layer; INL, inner nuclear layer; IPL, inner plexiform layer; ONL, outer nuclear layer; OPL, outer plexiform layer; PL, photoreceptor layer (scale bar: 50 μm.). **(B)** Measurement of retinal thickness from the ganglion cell layer (GCL) to the photoreceptor layer (PL); the number of cells per 500 μm in the GCL and the mean thicknesses of different retinal layers at 1 day of RIRI in rats are shown. **(C)** Apoptotic cells were detected by TUNEL assay in retinal sections at 1 day of RIRI in rats. Representative images of TUNEL-positive cells (green) are shown. The nuclei were counterstained with Hoechst 33342 (blue). Scale bar: 50 μm. **(D)** Bar graphs showing the average number of TUNEL-positive cells in the GCL, ONL, INL, and entire retina. The data are presented as the mean ± SEM (*P < 0.05, **P < 0.01, ***P < 0.001 vs. the control group; ^#^*p* < 0.05, ^##^*P* < 0.01, ^###^*P* < 0.001 vs. the HIOP group; *n* = 5 for each group). EA, Electroacupuncture.

To investigate whether EA can protect the retina from RIRI, we selected two pairs of acupoints for EA in rats, which were divided into the EA1 group [Jingming (BL1) + Shuigou (GV26)] and the EA2 group [Jingming (BL1) + Hegu (LI4)]. The two pairs of EA points were also used for the corresponding two sets of sham EA. H&E staining was used to observe the histopathological changes of the retina on the first day of RIRI (Figure 3A). Through measurement and statistical analysis of the entire retina, each layer of the retina, and the number of RGCs, we found that compared with the control group, the overall thickness of the retina and the thickness of each layer in the HIOP group and sham EA group showed an increasing tendency, but there were no significant differences compared to the EA group. The total retinal thickness and the thickness of each layer in the EA group were lower than in the HIOP group and the sham EA group. The number of RGCs in the HIOP group was lower than in the control group. However, no significant differences existed for any other groups (Figure 3B). Subsequently, TUNEL staining was applied to detect apoptosis of the retina (Figure 3C). The results showed that compared with the control group, every group except the sham group had a more significant increase in apoptotic cells. The number of apoptotic cells in the GCL and INL of the retina and the total apoptotic cells were significantly lower after EA treatment than in the HIOP group values. The number of apoptotic cells in the outer nuclear layer (ONL) was not markedly different in any group compared with the HIOP group (Figure 3D).

From the above results, we concluded that EA could protect the morphological structure of the retina on the first day of RIRI in rats and reduce the number of apoptotic cells in the retina.

### EA can activate DOR through the BDNF/TrkB pathway to inhibit RIRI-induced inflammatory factor production and increase the expression of anti-inflammatory factors on the first day of RIRI

**Figure 4:**
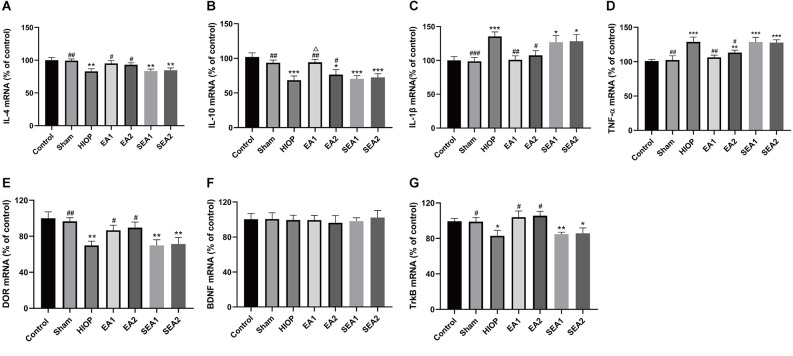
EA can activate DOR and elevate DOR and TrkB expression to reduce the inflammatory response at 1 day of RIRI. **(A–D)** The mRNA expression levels of proinflammatory cytokines (TNF-α and IL-1β) and anti-inflammatory cytokines (IL-4 and IL-10) were evaluated by RT–qPCR at 24 h of reperfusion following 1 h of retinal ischemia. **(E–G)** The mRNA expression levels of DOR, BDNF, and TrkB were evaluated by RT–qPCR at 24 h of reperfusion following 1 h of retinal ischemia. The data are expressed as the mean ± SEM (*n* = 6 for each group; *P < 0.05, **P < 0.01, ***P < 0.001 vs. the control group; ^#^*P* < 0.05, ^##^*P* < 0.01, ^###^*P* < 0.001 vs. the HIOP group; ^△^*P* < 0.05 vs. the EA2 group; *n* = 6 for each group). DOR, δ-opioid receptor; TrkB, tropomyosin-related kinase B.

The inflammatory response is one of the main mechanisms of RIRI and has critical effects on the pathological process of RIRI (Fang et al., 2015; Gonçalves et al., 2016). To further clarify whether EA can activate DOR in the rat retina and affect the production of proinflammatory and anti-inflammatory factors at 1 day of RIRI, RT–PCR was applied to detect the expression of associated factors. As shown in Figures 4A–D, the mRNA expression of the anti-inflammatory cytokine IL-4 was markedly decreased in the HIOP, SEA1, and SEA2 groups compared with the control group. The mRNA expression of the anti-inflammatory cytokine IL-10 was reduced in the HIOP, EA2, SEA1, and SEA2 groups compared with the control group. However, the decreases in IL-4 and IL-10 mRNA expression were attenuated in the EA1 and EA2 groups compared with the HIOP group. Specifically, the IL-10 mRNA expression of the EA1 group was higher than that of the EA2 group. The mRNA expression of the proinflammatory factor IL-1β was increased in the HIOP, SEA1, and SEA2 groups compared with the control group. TNF-α mRNA expression in the HIOP, EA2, SEA1, and SEA2 groups was elevated compared with that in the control group. After EA treatment, the mRNA expression of IL-1β and TNF-α in the EA1 and EA2 groups was dramatically decreased compared with that in the HIOP group. As shown in Figures 4E–G, DOR mRNA expression was strongly reduced in the HIOP, SEA1, and SEA2 groups compared with the control group. In contrast, the DOR expression in the EA1 and EA2 groups was elevated compared with that in the HIOP group. There was no significant difference in BDNF mRNA expression in any group. The mRNA expression of TrkB was reduced in the HIOP, SEA1, and SEA2 groups compared with the control group. The mRNA expression of TrkB in the EA1 and EA2 groups was increased compared with that in the HIOP group.

To sum up, these data informed that EA activated DOR and exerted anti-inflammatory effects through the BDNF/TrkB signaling pathway to suppress RIRI-induced generation of proinflammatory cytokines by prompting the release of anti-inflammatory cytokines on the first day of RIRI. Moreover, the EA1 group showed better release of the anti-inflammatory factor IL-10 than the EA2 group.

### EA can ameliorate injury and apoptosis in the retina at 3 days of RIRI in rats

**Figure 5:**
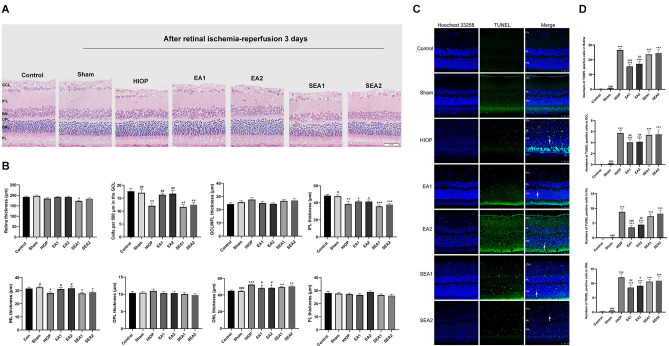
EA can ameliorate injury and apoptosis of the retina after RIRI for 3 days in rats. **(A)** H&E staining of retinal cross-sections showing the retinal tissue thickness of every group of ischemia-reperfusion (I/R) rats at 3 days of reperfusion. GCL, ganglion cell layer; INL, inner nuclear layer; IPL, inner plexiform layer; ONL, outer nuclear layer; OPL, outer plexiform layer; PL, photoreceptor layer (scale bar: 50 μm). **(B)** Measurement of retinal thickness from the ganglion cell layer (GCL) to the photoreceptor layer (PL); the number of cells per 500 μm in the GCL and the mean thickness of different retinal layers after RIRI for 3 days in rats are shown. **(C)** Apoptotic cells were detected by TUNEL assay in retinal sections at 3 days of ischemia-reperfusion injury in rats. Representative images of TUNEL-positive cells (green) are shown. The nuclei were counterstained with Hoechst 33342 (blue). Scale bar: 50 μm. **(D)** Bar graphs showing the average numbers of TUNEL-positive cells in the GCL, ONL, INL, and entire retina. The data are presented as the mean ± SEM (*P < 0.05, **P < 0.01, ***P < 0.001 vs. the control group; ^#^*p* < 0.05, ^##^*P* < 0.01, ^###^*P* < 0.001 vs. the HIOP group; *n* = 5 for each group).

To further investigate whether EA protects the retinas of rats from RIRI-induced HIOP on the 3rd day of RIRI, H&E staining and TUNEL staining were performed. A representative picture of H&E staining is shown in Figure 5A, and the statistics are displayed in Figure 5B. The statistics indicated that the total retinal thickness was not significantly different in any group. With regard to the number of RGCs, the numbers in the HIOP, SEA1, and SEA2 groups, but not in the EA1 and EA2 groups, were significantly decreased compared to the control group. The thickness of the IPL was reduced in the HIOP, EA1, EA2, SEA1 and SEA2 groups compared with the control group. The thickness of INL decreased in the HIOP, SEA1, and SEA2 groups compared with the control group, but after EA treatment, the thickness in the EA1 and EA2 groups was higher than in the HIOP group. Compared to the control group, the ONL thickness was increased in the HIOP, EA2, SEA1, and SEA2 groups. After EA treatment, the thickness of the ONL in the EA1 and EA2 groups was decreased. There were no significant differences in the changes of other layers. We also utilized TUNEL staining to visualize apoptotic cells in the retina at 3 days of RIRI. Representative pictures and statistics are shown in Figures 5C,D. Statistical analysis indicated that the number of apoptotic cells in the retina at 3 days of RIRI was significantly increased except in the sham group compared with the control group. However, the number of apoptotic cells in the EA1 and EA2 groups was significantly reduced compared to that in the HIOP group.

Our histopathology results indicated that EA ameliorated retinal injury and apoptosis of the retina after RIRI for 3 days in rats.

### EA can activate DOR through the BDNF/TrkB pathway to inhibit RIRI-induced proinflammatory cytokine production and increase the expression of anti-inflammatory factors on the 3rd day

**Figure 6:**
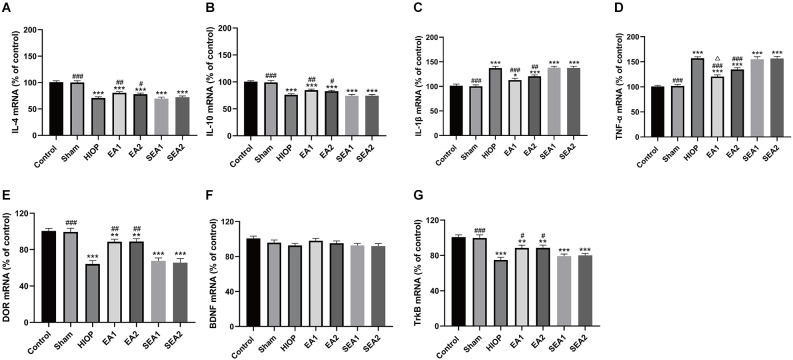
EA can activate DOR and elevate DOR and TrkB expression to reduce the inflammatory response after RIRI for 3 days. **(A–D)** The mRNA expression levels of proinflammatory cytokines (TNF-α and IL-1β) and anti-inflammatory cytokines (IL-4 and IL-10) were evaluated by RT–qPCR at 72 h of reperfusion following 1 h of retinal ischemia. **(E–G)** The mRNA expression levels of DOR, BDNF, and TrkB were evaluated by RT–qPCR at 72 h of reperfusion following 1 h of retinal ischemia. The data are expressed as the mean ± SEM (*P < 0.05, **P < 0.01, ***P < 0.001 vs. the control group; ^#^*P* < 0.05, ^##^*P* < 0.01, ^###^*P* < 0.001 vs. the HIOP group; ^△^*P* < 0.05 vs. the EA2 group; *n* = 6 for each group).

To further explore whether RIRI induces an inflammatory response and to investigate the therapeutic effect of EA over 3 days, the expression of the proinflammatory cytokines IL-1β and TNF-α and the anti-inflammatory cytokines IL-4 and IL-10 was analyzed using RT–qPCR (Figures 6A–D). The mRNA levels of IL-4 and IL-10 in the HIOP, EA1, EA2, SEA1, and SEA2 groups were dramatically decreased compared with those in the control group. However, the mRNA levels of IL-4 and IL-10 in the EA1 and EA2 groups showed increases after 3 days of EA treatment compared with the HIOP group. The expression of the proinflammatory cytokines IL-1β and TNF-α was increased in the HIOP, EA1, EA2, SEA1, and SEA2 groups compared with the control group. The mRNA expression levels of the proinflammatory cytokines IL-1β and TNF-α in the EA1 and EA2 groups were significantly lower than in the HIOP group. Furthermore, the expression of the proinflammatory cytokine TNF-α was lower in the EA1 group than in the EA2 group. The mRNA expression levels of DOR, BDNF, and TrkB are shown in Figures 6E–G. From these data, we determined that on the 3rd day of RIRI, the mRNA expression of DOR and TrkB was dramatically decreased in the HIOP, EA1, EA2, SEA1, and SEA2 groups. However, after EA treatment, DOR and TrkB mRNA expression was increased compared with that in the HIOP group. No significant changes in the mRNA levels of BDNF were observed in any group.

In summary, DOR was activated in the two EA groups, proinflammatory factors (IL-1β and TNF-α) were downregulated, and anti-inflammatory cytokines (IL-4 and IL-10) were upregulated through the BDNF/TrkB signaling pathway. Moreover, after 3 days of EA treatment, the EA1 group exhibited better inhibition of the proinflammatory cytokine TNF-α than the EA2 group.

### EA can ameliorate retinal injury and apoptosis at 7 days of RIRI in rats

**Figure 7:**
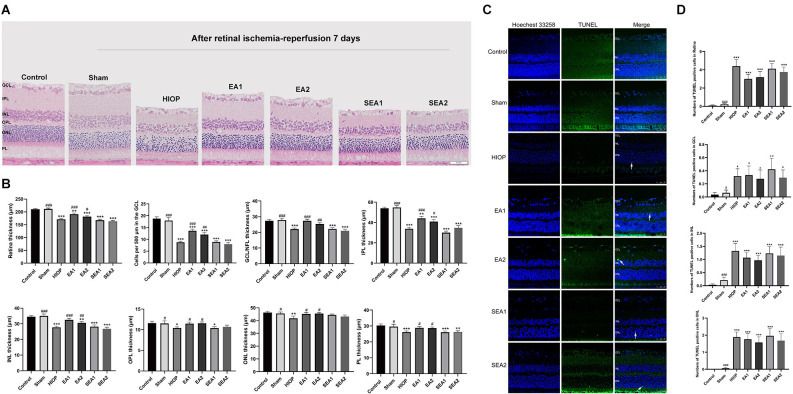
EA can ameliorate injury and apoptosis of the retina at 7 days of RIRI in rats. **(A)** H&E staining of retinal cross-sections showing the retinal tissue thickness of every group of RIRI rats at 7 days. GCL, ganglion cell layer; INL, inner nuclear layer; IPL, inner plexiform layer; ONL, outer nuclear layer; OPL, outer plexiform layer; PL, photoreceptor layer (scale bar: 50 μm). **(B)** Measurement of retinal thickness from the ganglion cell layer (GCL) to the photoreceptor layer (PL); the number of cells per 500 μm in the GCL and the mean thicknesses of different retinal layers after RIRI for 7 days in rats are shown. **(C)** Apoptotic cells were detected by TUNEL assay in retinal sections after RIRI for 7 days in rats. Representative images of TUNEL-positive cells (green) are shown. The nuclei were counterstained with Hoechst 33342 (blue). Scale bar: 50 μm. **(D)** Bar graphs showing the average numbers of TUNEL-positive cells in the GCL, ONL, INL, and entire retina. The data are presented as the mean ± SEM (*P < 0.05, **P < 0.01, ***P < 0.001 vs. the control group; ^#^*P* < 0.05, ^##^*P* < 0.01, ^###^*P* < 0.001 vs. the HIOP group; *n* = 5 for each group).

To determine whether EA can protect the retina at 7 days of RIRI, we also utilized H&E staining and TUNEL staining to observe retinal histopathology changes after RIRI for 7 days. A representative picture of H&E staining is shown in Figure 7A, and the statistics are displayed in Figure 7B. As shown in Figure 7A, after HIOP-induced RIRI for 7 days, the retinal thickness in the HIOP, EA1, EA2, SEA1, and SEA2 groups was decreased, especially in the GCL, IPL, and INL. And, the number of retinal cells in the GCL, INL, and ONL was reduced compared to the control group. Statistical analysis revealed that the total retinal thickness and the number of RGCs were significantly decreased in the HIOP, EA1, EA2, SEA1, and SEA2 groups compared with the control group, while after 7 days of EA treatment, the total retinal thickness and the number of RGCs were enormously increased compared with those in the HIOP group. The other layers of the retina presented similar statistical tendencies. Typical images of TUNEL staining and the statistics for apoptotic cells are shown in Figures 7C,D. The representative images show that there were significantly fewer TUNEL-positive cells on the seventh day than apoptotic cells on the first day and third day. Regardless of the total number of apoptotic cells or the numbers of apoptotic cells in the GCL, INL, and ONL, the apoptotic cell numbers were increased compared with those in the control group at 7 days of retinal ischemia-reperfusion.

These results demonstrate that EA can improve HIOP-induced RIRI on the 7th day, but it has little effect on preventing retinal cell apoptosis.

### EA can activate DOR and elevate DOR and TrkB expression to reduce the inflammatory response after RIRI for 7 days

**Figure 8:**
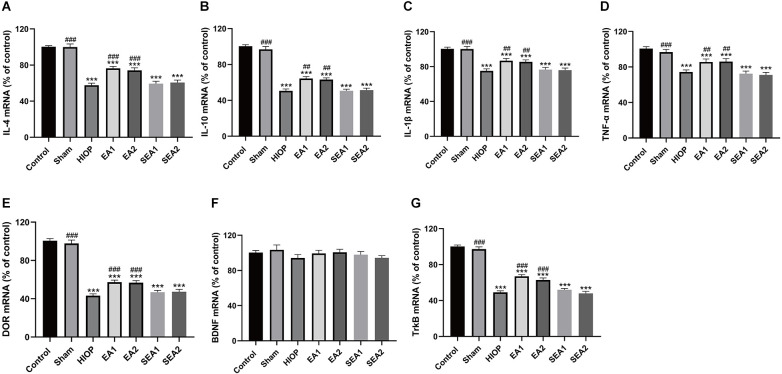
EA can activate DOR and elevate DOR and TrkB expression to reduce the inflammatory response after RIRI for 7 days. **(A–D)** The mRNA expression levels of proinflammatory cytokines (TNF-α and IL-1β) and anti-inflammatory cytokines (IL-4 and IL-10) were evaluated by RT–qPCR at 7 days of reperfusion following 1 h of retinal ischemia. **(E–G)** The mRNA expression levels of DOR, BDNF, and TrkB were evaluated by RT–qPCR at 7 days of reperfusion following 1 h of retinal ischemia. The data are expressed as the mean ± SEM (***P < 0.001 vs. the control group; ^##^*P* < 0.01, ^###^*P* < 0.001 vs. the HIOP group; *n* = 6 for each group).

To further clarify whether EA can activate DOR in the rat retina and affect the production of proinflammatory and anti-inflammatory factors through the BDNF/TrkB pathway at 7 days of RIRI, RT–PCR was performed to evaluate the expression of related factors. As shown in Figures 8A–D, the mRNA expression levels of the anti-inflammatory molecules IL-4 and IL-10 and the pro-inflammatory molecules IL-1β and TNF-α were significantly decreased in the HIOP, EA1, EA2, SEA1, and SEA2 groups compared with the control group. After 7 days of EA treatment, the mRNA expression of the anti-inflammatory molecules IL-4 and IL-10 and the pro-inflammatory molecules IL-1β and TNF-α in the EA1 and EA2 groups was increased compared with that in the HIOP group. The mRNA levels of DOR were dramatically decreased in the HIOP, EA1, EA2, SEA1, and SEA2 groups after HIOP-induced RIRI on the 7th day compared with the control group. The mRNA expression of DOR was increased in the EA1 and EA2 groups that underwent EA treatment for 7 days compared with the HIOP group. A similar tendency was shown in the mRNA expression of TrkB. After HIOP-induced RIRI on the seventh day, the mRNA levels of TrkB were significantly decreased in the HIOP, EA1, EA2, SEA1, and SEA2 groups compared with the control group. However, after 7 days of EA treatment, the mRNA levels of TrkB were increased in the EA1 and EA2 groups compared with the HIOP group (Figures 8E–G).

All these results demonstrate that the activation of DOR can regulate correlative proinflammatory and anti-inflammatory factors to protect the retina after 7 days of RIRI through the BDNF/TrkB signaling pathway.

## Discussion

Our study focused on the protective effect of EA against RIRI in rats and its related mechanisms. According to the above data, there are three main findings and implications. First, HIOP-induced RIRI causes damage to the retina and apoptosis of retinal cells in rats at 1, 3, 5, and 7 days. Second, EA has a protective effect on the retina at 1, 3, and 7 days of RIRI and can reduce the apoptosis of retinal cells on the first and third day in rats. Third, the protective effect of EA against RIRI in rats occurs mainly through DOR-BDNF/TrkB signaling to reduce the levels of proinflammatory factors and increase the levels of anti-inflammatory factors.

The HIOP model in rats imitates circulatory disturbance of the retina resulting from elevated intraocular pressure followed by compression of retinal blood vessels and gives rise to temporary interruption of retinal blood flow (Li et al., 2018). The subsequent natural reperfusion causes retinal inflammation, oxidative stress, glutamate excitotoxicity, and apoptosis, which is the vital pathophysiological basis of various ischemic retinal diseases, such as DR, RVO, and glaucoma (Gao et al., 2014; Minhas et al., 2016). It can be seen from the results of our study that in the early stages of retinal ischemia-reperfusion, the total retinal thickness increases and reaches a peak within 3 days. Some scholars have proposed that retinal thickness increases in the early stage of RIRI, which may be caused by the activation of retinal glial cells, which breaks the homeostasis of retinal penetration (Reichenbach et al., 2007). Reports suggest that the increase in retinal thickness at the early stage of RIRI is closely related to aquaporin-4 (AQP-4; Da and Verkman, 2004; Iandiev et al., 2006). From 3 to 7 days of RIRI, the total thickness of the retina and the number of RGCs continued to decrease over time, and the entire retinal structure was destroyed, which is consistent with the findings of previous studies (Zhang et al., 2014). In the TUNEL experiment, with the passage of I/R time, TUNEL-positive cells started to appear from the innermost GCL, IPL, and INL and gradually progressed to the ONL and OPL layers. Other scholars have reported similar findings (Shibuki et al., 2002; Zheng et al., 2004).

EA, a characteristic of traditional Chinese medicine therapy, has been widely used for ophthalmological diseases. Previously, some scholars have revealed that EA treatment had preserved retinal function in rats with experimental glaucoma (Chan et al., 2005). In the present study, we selected the most frequently used two pairs of visual-related acupuncture points, the EA1 pair [Jingming (BL1) + Shuigou (GV26)] and the EA2 pair [Jingming (BL1) + Hegu (LI4)]. Since applied EA at the Jingming (BL1) acupoint can improve conjunctival microcirculation and protect visual function in rabbits with HIOP (Zhang, 2008). Applying EA to the Shuigou (GV26) acupoint increases blood flow in the retina and protects the visual acuity of patients with central retinal artery occlusion (Shi et al., 2015). EA also improves flash electroretinogram in rabbits with unilateral optic nerve transection by stimulating at the Hegu (LI4), and this effect has the specificity of EA acupoints (Liu et al., 2005). Therefore, we combined the above three acupuncture points into two pairs to observe the therapeutic effect and compare whether there is any difference in the therapeutic effect. As shown in the images of H&E staining at 1, 3, and 7 days of RIRI, the two pairs of EA points had a protective impact on the retinal structure and RGC numbers. The results of TUNEL staining revealed that the numbers of apoptotic cells in the two EA groups at 1 and 3 days of RIRI were significantly reduced, consistent with the previous studies (Wang et al., 2020; Xing et al., 2021). But there was no significant effect on the seventh day of RIRI because the peak of apoptosis had passed, leading to reduction in the number of apoptotic cells in each group.

IL-1β and TNF-α are pivotal inflammatory factors in the pathological process of RIRI (Osborne et al., 2004). A recent report showed that EA could treat brain I/R injury by inhibiting the inflammatory reaction of the brain and exerting a neuroprotective effect (Long et al., 2019). In our study, on the first and third day of RIRI, the levels of the proinflammatory factors IL-1β and TNF-α were decreased after EA treatment. In contrast, the levels of the anti-inflammatory factors IL-4 and IL-10 increased in the two EA groups. Specifically, the IL-10 expression level of the EA1 group was higher than that of the EA2 group at 1 day of RIRI, and the TNF-α expression level of the EA1 group was significantly lower than that of the EA2 group on the third day of RIRI. Although stimulation of the two pairs of acupoints with EA reduced the levels of proinflammatory factors and increased the levels of anti-inflammatory factors after RIRI, we conclude that the effect of EA1 was better than that of EA2, possibly due to stimulation of the Shuigou (GV26) acupoint with EA. Our previous studies and other scholars’ studies have demonstrated that Shuigou (GV26) has a better effect on the inflammation inhibition of ischemia-reperfusion injury (Zhang et al., 2010; Geng et al., 2020).

However, in the late stage of RIRI, the expression levels of IL-1β and TNF-α decreased, consistent with the previous reports (Hangai et al., 1995; Berger et al., 2008; Kim et al., 2017). Research has suggested that this may be caused by the difference in the ratio of TNF receptor 1 or TNF receptor 2 in the late stage of RIRI or a caspase-dependent mechanism (Berger et al., 2008). The functions of TNF receptor 1 and TNF receptor 2 are different. TNF receptor 1 can activate Bcl-2 family members, caspase family members, and reactive oxygen species, which trigger subsequent inflammatory reactions and induce RGC apoptosis (Zimmermann and Green, 2001). The mechanism of high expression of inflammatory factors at the initial stage of RIRI may be that TNF-α is mainly used by TNF-receptor 1 to exert its proinflammatory and proapoptotic effects (Agarwal and Agarwal, 2012). The primary function of TNF receptor 2 is to activate the c-Jun N-terminal kinase (JNK) and nuclear factor kappa B (NF-κB) pathways and interfere with the process of apoptosis, thereby protecting RGCs (Agarwal and Agarwal, 2012). Therefore, in the late stage of RIRI, the expression of TNF-α in the two EA groups was higher than in the HIOP, SEA1, and SEA2 groups. We believe that EA may increase the expression of TNF receptor 2 in the late stage of injury, which has a protective effect on the retina. In short, EA treatment helps alleviate retinal damage after RIRI in response to inflammation.

The destruction of retinal integrity caused by RIRI is often accompanied by impairment of visual function (Li et al., 2021). To our knowledge, there are few studies on the treatment of visual dysfunction caused by RIRI with EA. However, both basic science research and clinical studies support the hypothesis that EA and/or acupuncture may benefit visual function in patients with retinitis pigmentosa and that the mechanism through which EA might exert its action on the regulation of NGF and BDNF and/or their receptors in retinal cells (Pagani et al., 2006; Bittner et al., 2014). The present study provides evidence that EA activates DOR to fight against inflammation and protects against degeneration of RGCs and damage to the retinal structure through BDNF and its receptor TrkB. Therefore, EA can also alleviate the visual dysfunction caused by RIRI through DOR-BDNF/TrkB pathway and ultimately improve the visual acuity of rat.

Studies have shown that acupuncture can affect the mRNA and protein expression of DOR and TrkB in different tissues, and the expression levels of mRNA are consistent with that at the protein level (Li et al., 2020; Liu et al., 2022). Therefore, we only detected the expression of the mRNA level but not the protein level in the present study. Our results demonstrate that EA increased the mRNA expression of DOR and TrkB after RIRI, but there was no significant difference in the level of BDNF mRNA in the early and late stages of RIRI. We speculate that the reason why BDNF did not decrease during the RIRI was due to positive feedback, anterograde transport, and activated microglia, just like in hypoxic cerebral injury (Tian et al., 2013a). There are two main isoforms of TrkB: the full-length form (TrkB-FL) and the truncated form (tTrkB; Vidaurre et al., 2012). Ischemia can easily cause downregulation of TrkB-FL, and activation of DOR can reverse this result in our past research (Tian et al., 2013b). Therefore, we believe that the maintenance of normal levels of BDNF expression is a result of multiple factors in the ischemic retina and the significant change of TrkB was mainly represented by the BDNF/TrkB pathway. In addition, our previous research has demonstrated that EA can activate DOR and exert a neuroprotective effect *via* the BDNF-TrkB pathway after brain I/R (Geng et al., 2020). In our study, EA may activate the DOR-BDNF/TrkB pathway and exert a protective effect against RIRI to improve retinal pathological damage in rats.

## Conclusion

In summary, our data show that the protective effect of EA might depend on the repression of the inflammatory response through the DOR-BDNF/TrkB signaling pathway after RIRI. The results in our study above affirm that EA at Shuigou (GV26) and Jingming (BL1) or at Hegu (LI4) and Jingming (BL1) can provide a reference for the clinical treatment of RIRI, especially EA at Shuigou (GV26) and Jingming (BL1), which was significantly more effective in controlling inflammation.

## Data Availability Statement

The original contributions presented in the study are included in the article/supplementary material, further inquiries can be directed to the corresponding author/s.

## Ethics Statement

The animal study was reviewed and approved by the Experimental Animal Ethics Committee of Shanghai University of Traditional Chinese Medicine.

## Author Contributions

RG contributed by performing most of the experiments and writing the manuscript. YZha assisted in completing all experiments. NJ and JJ revised the manuscript. HX performed histopathological evaluation and wrote the histopathological results section of the manuscript. XT conceived and designed the study, revised the manuscript and secured most of the funding. The other authors undertook part of the work during the experiment. All authors contributed to the article and approved the submitted version.
